# Ill Effects of Tight Bandaging

**Published:** 1858-12

**Authors:** 


*Ill Effects of Tight Bandaging.*—Dr. Dalton, of Aberdeen, in a letter to the Editor of the Buffalo Medical Journal, relates a case in which a negro boy, aged sixteen, lost his leg from the effects of tight bandaging in a case of fracture. Dry gangrene followed, and amputation was rendered necessary. A somewhat similar case occured in our own practice not long since, in which an ignorant practitioner, in treating a fracture of the arm in the case of an interesting little girl, applied the bandage so tight over a rude splint, as to cause the death of the part. On our removing the bandage a week after its application, gangrene and death of the arm was manifest, and at our request, amputation was performed by Professor Pope. Young surgeons should be especially careful in the application of bandages to see that they are not too tight.—*St. Louis Medical & Surgical Journal.*

